# The inhibitory receptor LAG3 affects NK cell IFN-γ production through glycolysis and the PSAT1/STAT1/IFNG pathway

**DOI:** 10.1128/mbio.00230-25

**Published:** 2025-04-29

**Authors:** Hongchi Ge, Nan Guo, Yufei Liu, Bin Lang, Xiaowan Yin, Xiaowen Yu, Zining Zhang, Yajing Fu, Haibo Ding, Qinghai Hu, Xiaoxu Han, Wenqing Geng, Hong Shang, Yongjun Jiang

**Affiliations:** 1 State Key Laboratory for Diagnosis and Treatment of Infectious Diseases, NHC Key Laboratory of AIDS Prevention and Treatment, National Clinical Research Center for Laboratory Medicine, The First Hospital of China Medical University, China Medical University 159407, Shenyang, Liaoning, China; University of California, Davis, Davis, California, USA

**Keywords:** LAG3, HIV, NK, IFN-γ, glycolysis, STAT1

## Abstract

Natural killer (NK) cells are integral to the innate immune system and crucial for antiviral defense. NK cell activation and functional state are suppressed by inhibitory receptors. Lymphocyte activation gene 3 (LAG3) is an important inhibitory receptor, but the associated signaling pathways that regulate lymphocyte function remain to be elucidated. In addition, the effect of LAG3 on NK cell function during HIV infection and its specific mechanisms are unclear. In this study, we observed that LAG3 expression by NK cells is elevated in HIV-infected individuals and inversely correlated with CD4/CD8 ratio and CD4^+^ T cell count. LAG3+ NK cells produce lower levels of interferon-gamma (IFN-γ), but LAG3-Fc protein significantly enhances NK cell function. The activation of LAG3 significantly inhibits IFN-γ production and Ki67 expression by NK cells. Our transcriptome sequencing and *in vitro* data show for the first time that LAG3 not only regulates the transcription of MYC and several glycolysis-related enzyme genes via the PI3K/AKT/mTOR signaling pathway to inhibit glycolysis in NK cells but also suppresses the STAT1/IFNG pathway by upregulating PSAT1 expression, thus limiting IFN-γ production by NK cells via these two different pathways. Overall, these results provide new insights and identify potential targets for immunotherapy of HIV infection.

**IMPORTANCE:**

We demonstrate that lymphocyte activation gene 3 (LAG3) expression is upregulated on natural killer (NK) cells during HIV infection. LAG3 inhibits glycolysis in NK cells and also upregulates PSAT1 expression to suppress activation of the STAT1/IFNG pathway, thus restricting interferon-gamma production by NK cells. These results provide new clues to study the effects of LAG3 on the metabolism and functional exhaustion of NK cells and offer a potential target for the treatment of HIV.

## INTRODUCTION

Natural killer (NK) cells play a significant role in the immune system by mediating the control of viral infections and the proliferation of malignant cells (1–3). The activation status and function of NK cells are regulated by the expression of multiple inhibitory receptors (4). As an inhibitory receptor, lymphocyte activation gene 3 (LAG3) is expressed on the surface of many kinds of immune cells, such as conventional T cells, regulatory T cells, and NK cells (5, 6). LAG3 is also contained in lysosomes (7). The binding of LAG3 to its corresponding ligands leads to immune inhibition, playing an important role in the regulation of immune homeostasis and the functions of effector immune cells (8). Upon T cell activation, LAG3 undergoes rapid translocation from lysosomes to the cell surface, where it inhibits T cell functions (9). Increased LAG3 expression in tumor-infiltrating lymphocytes (TILs) has been reported in hepatocellular carcinoma, and blockade of the binding of LAG3 to its ligand, MHC-II, leads to an increase in the number of TILs and enhances their ability to secrete cytokines, thus promoting anti-tumor immunity (10). In autoimmune diseases, blockade or knockout of the LAG3 gene can accelerate disease progression (11–14).

A study of NK cells using two mouse tumor models reported that interleukin (IL)-12 combined with LAG3 blockade reduced the metastasis of tumor cells and upregulated the number and function of NK cells, suggesting that the regulatory effect of LAG3 on NK cells might be similar to the effect on T cells (8). However, there is a scarcity of studies investigating the impact of LAG3 on NK cells in human tumors and chronic infections. In particular, the signaling pathways by which LAG3 regulates the function of T cells and NK cells in tumors and infections remain unknown. During HIV infection, the phenotype and subset distribution of NK cells change dramatically. However, no additional reports describing the effect of LAG3 on NK cell function during HIV infection or the associated intracellular mechanisms have been published.

In this study, we examined the expression of LAG3 in populations of total NK cells and three NK cell subsets. In particular, we analyzed the correlation between LAG3 and HIV disease progression and further investigated the effect of LAG3 on various NK cell functions. LAG3 expressed on NK cells was activated using LAG3-activating antibodies, and transcriptome sequencing analyses of these cells were conducted to identify the relevant mechanistic pathways. Finally, *in vitro* experiments confirmed that LAG3 suppresses the function of NK cells through two pathways. Taken together, our findings provide new insights that could aid in the development of novel immunotherapeutic strategies for treating HIV.

## RESULTS

### LAG3 expression on NK cells is elevated in HIV-infected individuals and correlated with HIV disease progression

**Fig 1:**
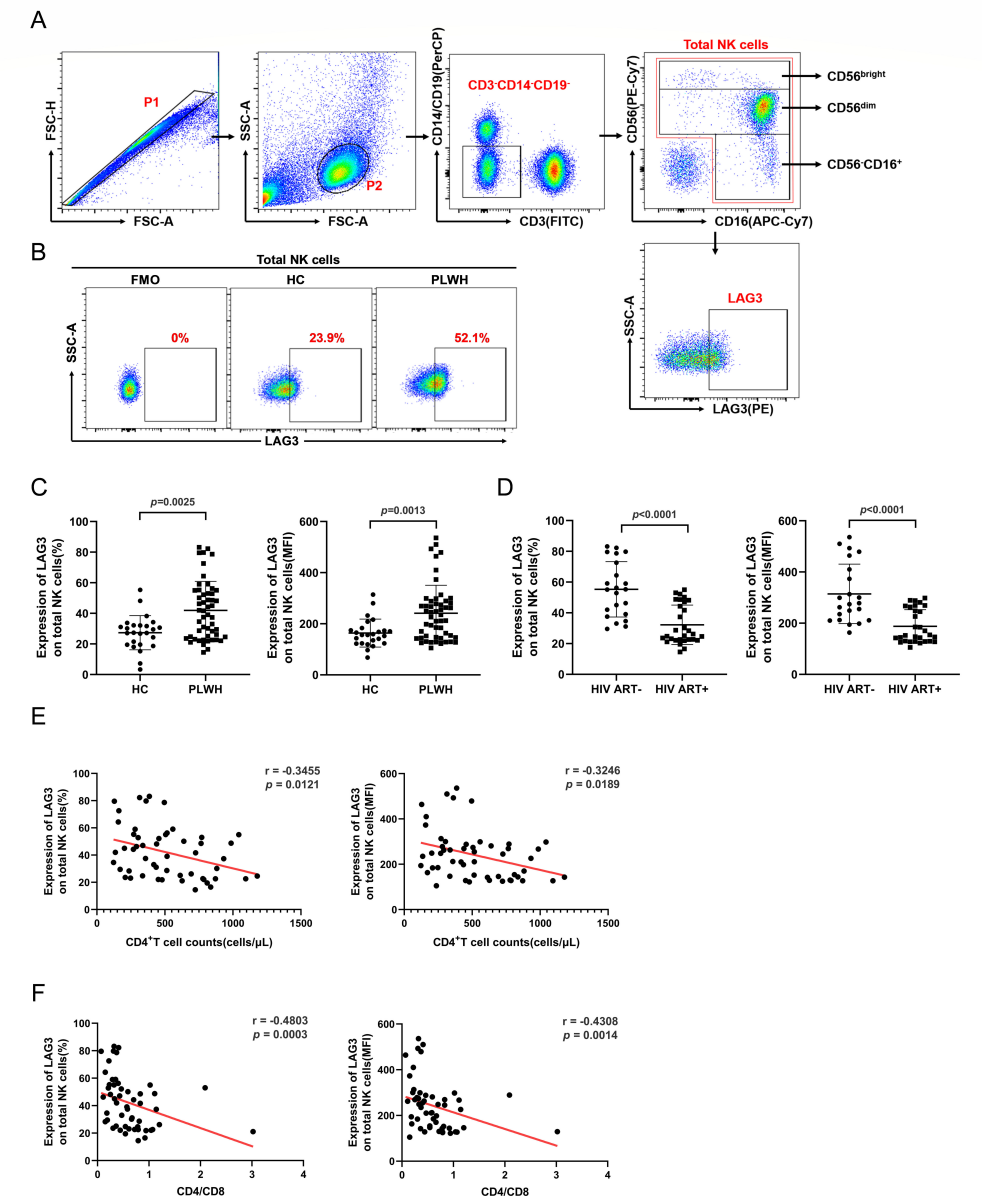
LAG3 expression on NK cells is elevated in HIV-infected individuals and correlated with HIV disease progression. (**A**) Flow cytometry gating strategy used for identification of NK cells and subsets. (**B**) Representative flow cytometry plots indicating the expression of LAG3 on NK cells in the HC and PLWH groups. Fluorescence minus one (FMO) control was applied to establish the gating threshold. (**C**) Comparison of the percentages (left) and MFI (right) of LAG3 on total NK cells from the HC (*n* = 25) and PLWH (*n* = 52) groups. (**D**) Comparison of the percentages (left) and MFI (right) of LAG3 on total NK cells from the HIV ART− (*n* = 22) and HIV ART+ (*n* = 30) groups. All 30 ART-treated individuals were on ART for more than 2 years and had undetectable plasma HIV RNA (viral load; VL). (**E and F**) Spearman correlation analysis of absolute CD4^+^ T cell count (cells/µL) and CD4/CD8 ratio with LAG3 expression (left: percentage; right: MFI) on NK cells (*n* = 52).

LAG3 expression levels were determined for total NK cells and three NK cell subsets isolated from 52 people living with HIV (PLWH; including 22 untreated and 30 antiretroviral therapy [ART] treated) and 25 healthy controls (HCs; Fig. 1A and B). The 30 ART-treated patients had been on treatment for over 2 years and exhibited undetectable viral loads (HIV RNA, <20 copies/mL). LAG3 expression levels and mean fluorescence intensity (MFI) on total NK cells from PLWH were higher compared with cells from HCs (*P* = 0.0025; *P* = 0.0013; Fig. 1C). LAG3 expression decreased significantly after ART (%: *P* < 0.0001; MFI: *P* < 0.0001; Fig. 1D). We found similar results on NK cell subsets (Fig. S1A and C). The expression of LAG3 was the highest on CD56^bright^ NK cells from HIV ART− and the lowest on CD56^−^CD16^+^ NK cells from HC and PLWH groups (Fig. S1B and D). Unfortunately, we did not observe any significant differences in LAG3 expression levels between immunological responders, defined as those with a CD4^+^ T cell count >500 cells/µL, and immunological non-responders, characterized by a CD4^+^ T cell count <350 cells/µL after more than 2 years of ART (Fig. S1E).

As shown in Fig. 1E and F, LAG3 expression on NK cells from PLWH was negatively correlated with absolute CD4^+^ T cell count (%: *r* = −0.3455, *P* = 0.0121; MFI: *r* = −0.3246, *P* = 0.0189) and CD4/CD8 ratio (%: *r* = −0.4803, *P* = 0.0003; MFI: *r* = −0.4308, *P* = 0.0014). No significant correlation was found between LAG3 expression and viral load in PLWH who did not receive ART (Fig. S1F). Similar results were obtained in analyses of NK cell subsets (Fig. S1G through I). These results indicate that LAG3 expression on NK cells may serve as a predictor of HIV progression.

### LAG3+ NK cells exhibit reduced activation and IFN-γ production during HIV infection

**Fig 2:**
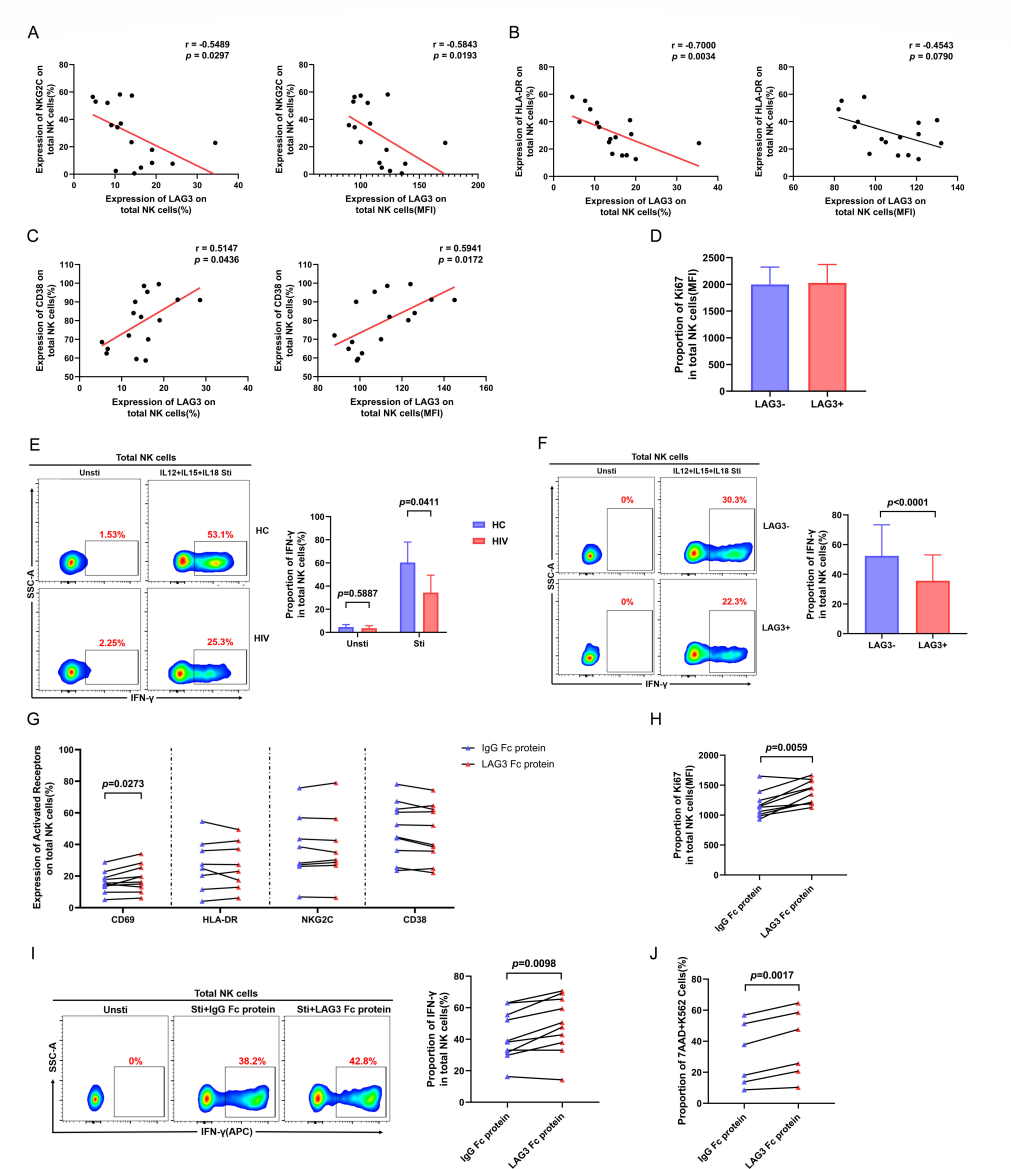
High LAG3 expression inhibits NK cell activation and interferon-gamma (IFN-γ) production, and LAG3-Fc protein significantly enhances NK cell function. (**A–C**) Analysis of the correlation between NKG2C, HLA-DR, and CD38 expression and LAG3 expression (left: percentage; right: MFI) on NK cells from HIV groups (*n* = 16). (**D**) Paired comparisons of Ki67 in LAG3+ and LAG3− NK cells from HIV groups (*n* = 15). (**E**) Comparison of IFN-γ production by NK cells from the HIV-infected (*n* = 6) and HC (*n* = 6) groups. (**F**) Paired comparisons were performed between IFN-γ producing LAG3+ and LAG3− NK cells following 24 h stimulation with the cytokine mixture including IL-12 (10 ng/mL), IL-15 (50 ng/mL), and IL-18 (100 ng/mL) for stimulated groups (*n* = 29). For unstimulated groups, peripheral blood mononuclear cells (PBMCs) were cultured without the cytokine mixture. (**G and H**) Paired comparisons of CD69, HLA-DR, NKG2C, CD38, and Ki67 expression on NK cells from HIV groups (CD69: *n* = 10, HLA-DR: *n* = 8, NKG2C: *n* = 8, CD38: *n* = 10, Ki67: *n* = 10) after treatment with 2 µg/mL LAG3-Fc or IgG-Fc protein (as a negative control). (**I**) Paired comparison of the production of IFN-γ in NK cells from HIV groups (*n* = 10) after treatment with 2 µg/mL LAG3-Fc or IgG-Fc protein. (**J**) Paired comparison of cytotoxicity of NK cells against K562 targets in HC group (*n* = 6) following 24 h treatment with 2 µg/mL LAG3-Fc or IgG-Fc protein.

To determine whether upregulation of LAG3 expression affects the function of NK cells, we first examined NK cell expression of activated receptors (NKG2C, human leukocyte antigen DR [HLA-DR], and CD38) and Ki67, which is a highly sensitive nuclear marker of cell proliferation. The expression of NKG2C and HLA-DR on NK cells in HIV groups was negatively correlated with LAG3 level (NKG2C: %: *r* = −0.5489, *P* = 0.0297; MFI: *r* = −0.5843, *P* = 0.0193; HLA-DR: %: *r* = −0.7000, *P* = 0.0034; Fig. 2A and B), and CD38 expression was positively correlated with LAG3 level (%: *r* = 0.5147, *P* = 0.0436; MFI: *r* = 0.5941, *P* = 0.0172; Fig. 2C). We did not find a significant difference in Ki67 expression between LAG3+ and LAG3− NK cells (Fig. 2D).

NK cells from HIV groups exhibited lower interferon-gamma (IFN-γ) production (*P* = 0.0411; Fig. 2E). IFN-γ production was lower in LAG3+ NK cells than LAG3− NK cells (*P* < 0.0001; Fig. 2F). Therefore, we speculated that the upregulation of LAG3 expression in HIV groups promotes the functional depletion of NK cells by inhibiting their activation and IFN-γ production.

We also examined whether blockade of the interaction between LAG3 and its ligands could enhance the function of NK cells. Recombinant human LAG3-Fc protein was used to block ligand binding during *ex vivo* stimulation for 24 h. Significant increases in CD69 and Ki67 expression as well as IFN-γ production were observed (CD69: *P* = 0.0273; Fig. 2G; Ki67: *P* = 0.0059; Fig. 2H; IFN-γ: *P* = 0.0098; Fig. 2I), but HLA-DR, NKG2C, and CD38 expression did not significantly change (Fig. 2G). Collectively, these results indicate that the LAG3-Fc protein mitigates the inhibitory effect of LAG3 on NK cell function.

To assess whether a physiologically relevant level of stimulation influences NK cell functionality subsequent to LAG3 ligand blockade, we pretreated NK cells with LAG3 Fc and IgG Fc proteins for a duration of 2 h and thereafter evaluated their capacity to kill K562 cells. Our results indicated that the LAG3 Fc protein augmented the cytotoxicity of NK cells toward K562 cells, exhibiting a significantly elevated level of killing compared to NK cells pretreated with IgG Fc protein (*P* = 0.0017; Fig. 2J).

### Activation of LAG3 significantly inhibits IFN-γ production and Ki67 expression in NK cells

**Fig 3:**
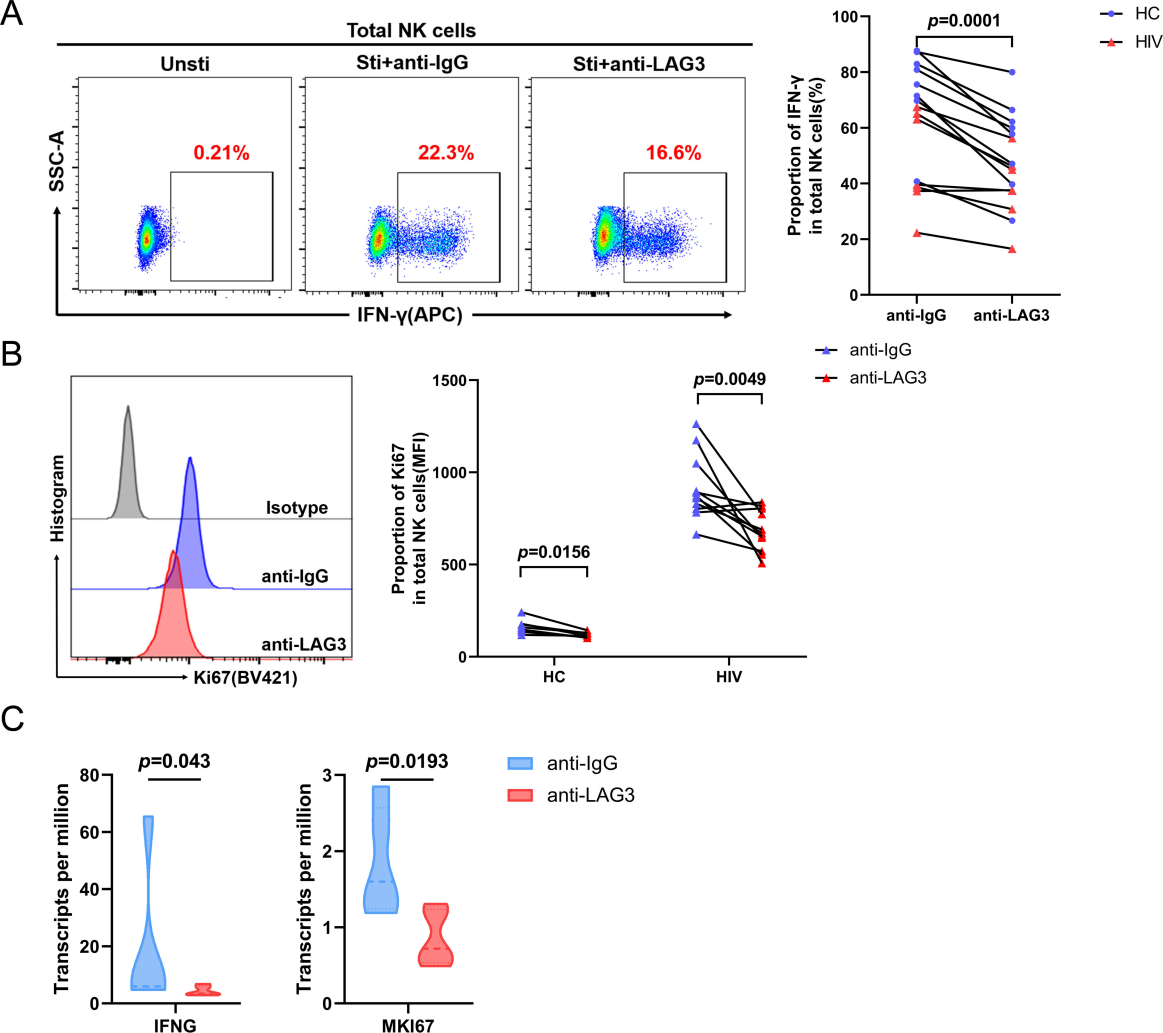
LAG3 activation significantly inhibits IFN-γ production and Ki67 expression in NK cells. (**A and B**) Paired comparisons of the IFN-γ production and Ki67 expression in NK cells after treatment with 10 µg/mL LAG3 antibody or IgG control from the HC and HIV groups (IFN-γ: HC, *n* = 8, HIV, *n* = 7; Ki67: HC, *n* = 7, HIV, *n* = 11). (**C**) Paired comparisons of IFNG and MKI67 TPM expression in NK cells after the above treatment in cells from the HC group (*n* = 5).

Next, we evaluated the effect of LAG3 activation on the function of NK cells. First, the optimal concentration of anti-LAG3 antibody (10 µg/mL; Fig. S2A) was determined, and the antibody was then used to activate LAG3. Following LAG3 activation, IFN-γ production and NK cell proliferation decreased significantly in both HC and HIV groups (IFN-γ: *P* = 0.0001; Ki67: HC, *P* = 0.0156, HIV+, *P* = 0.0049; Fig. 3A and B). The expression of mRNAs (transcripts per million, TPM) specific to the IFNG and MKI67 genes, which encode IFN-γ and Ki67, respectively, also decreased after LAG3 activation in transcriptome sequencing analysis described later in detail (Fig. 3C). The above results suggest that LAG3 activation inhibits the function of NK cells, thus highlighting the importance of studying changes in related intracellular signaling pathways.

### Transcriptome sequencing showed that LAG3 inhibits glycolysis-related gene expression in NK cells

**Fig 4:**
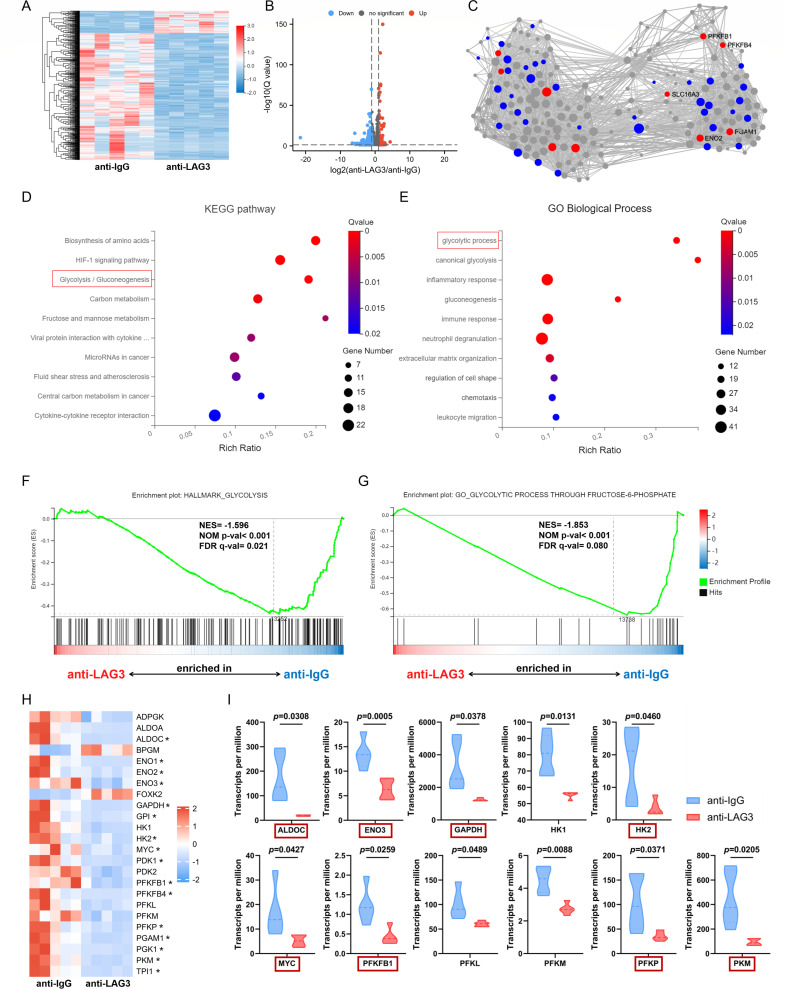
Transcriptome sequencing showed that LAG3 inhibits glycolysis-related gene expression in NK cells. (**A**) Heatmap of transcriptome sequencing data (anti-IgG: *n* = 5, anti-LAG3: *n* = 5); blue indicates downregulated gene expression (row *Z*-score  < 0), and red indicates upregulated gene expression (row *Z*-score > 0). (**B**) Volcanic map of all identified genes and DEGs. (**C**) Gene subnetworks and top network key drivers of downregulated DEGs. KDA genes are indicated in red, initial genes in blue, and all other genes in gray. (**D and E**) KEGG pathway and GO biological process enrichment analyses of downregulated DEGs. (**F and G**) GSEA of enriched gene sets in the anti-LAG3 and anti-IgG groups. (**H**) Heatmap of differentially expressed glycolysis-related genes. (**I**) Paired comparisons of glycolysis-related gene expression values in NK cells from the anti-LAG3 (*n* = 5) and anti-IgG (*n* = 5) groups.

To identify the intracellular signal pathways through which LAG3 regulates the function of NK cells, NK cells were cultured *in vitro* for 24 h with anti-LAG3 and anti-IgG antibodies and then subjected to transcriptome sequencing analysis. A total of 757 differentially expressed genes (DEGs) were identified. As shown in Fig. 4A and B, after anti-LAG3 treatment of NK cells, 113 genes were upregulated, and 644 genes were downregulated. There was a significant difference in the expression of these DEGs between the two groups. Potential interactions between the common downregulated DEGs were identified using key driver analysis (KDA; Fig. 4C). Among the top 10 key genes, 5 genes were related to cellular glucose metabolism, which strongly suggests that LAG3 activation alters glucose metabolism in NK cells. Kyoto Encyclopedia of Genes and Genomes Analysis (KEGG) pathway and Gene Ontology (GO) biological process enrichment analyses showed that the downregulated DEGs were significantly concentrated in the glycolysis/gluconeogenesis (Fig. 4D) and glycolysis process categories (Fig. 4E). Gene set enrichment analysis (GSEA) showed that genes related to glycolysis (Fig. 4F) and glycolytic processes through fructose-6-phosphate (Fig. 4G) were upregulated primarily in control NK cells, suggesting that LAG3 activation downregulates glycolysis in NK cells. A heatmap of the DEGs (Fig. 4H) showed that several glycolysis-related enzyme genes were significantly downregulated after anti-LAG3 treatment, and the expression of these genes decreased significantly after LAG3 activation (Fig. 4I).

### *In vitro* experiments indicated that LAG3 regulates NK cell function by inhibiting glycolysis via the PI3K/AKT/mTOR signaling pathway

**Fig 5:**
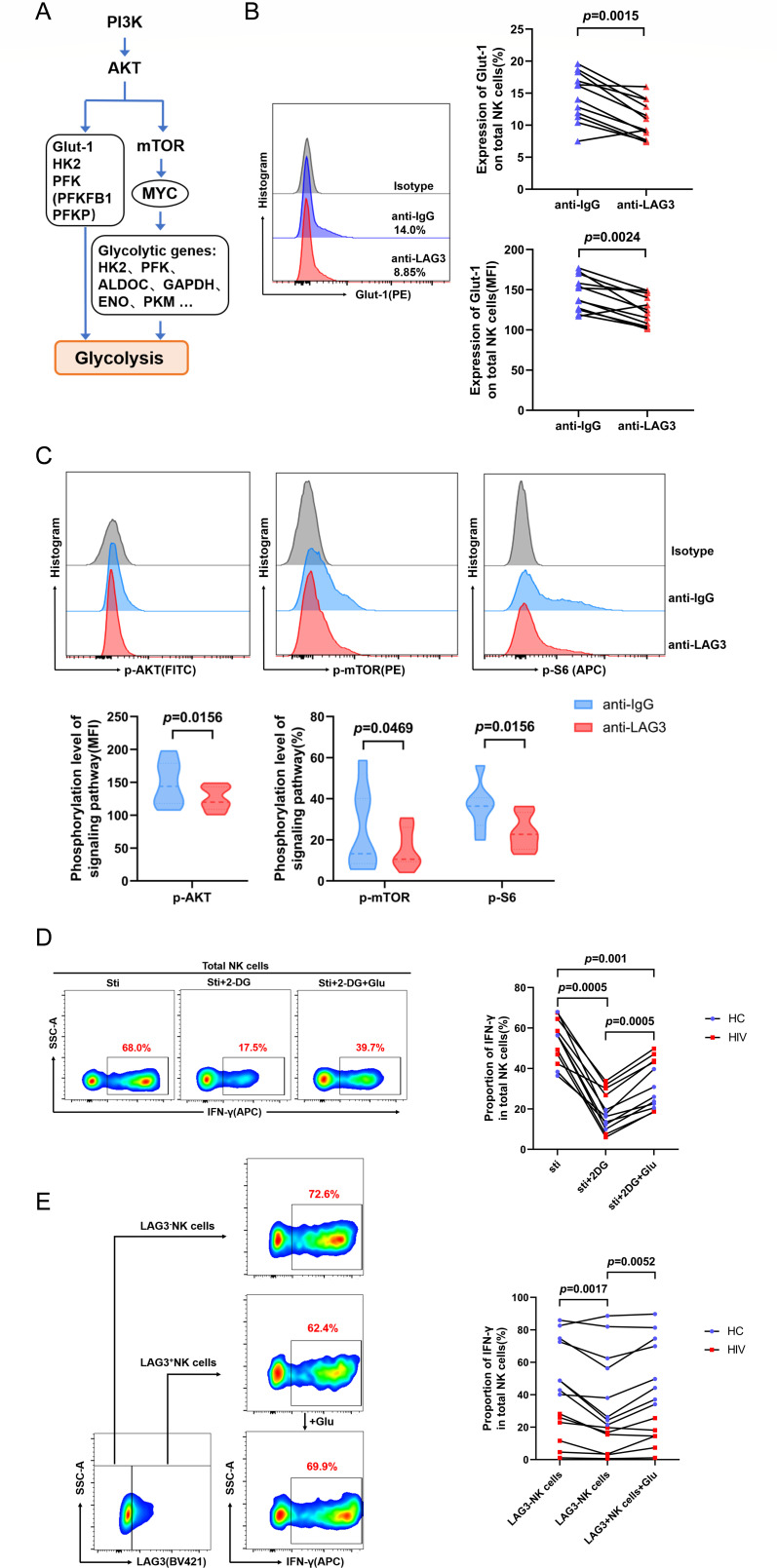
*In vitro* experiments demonstrated that LAG3 inhibits glycolysis in NK cells via the PI3K/AKT/mTOR signaling pathway, thereby affecting NK cell function. (**A**) Schematic diagram of the PI3K/AKT signaling pathway regulating glycolysis in NK cells. (**B**) Paired comparisons of Glut-1 expression on NK cells from the HIV group (*n* = 12) after treatment with 10 µg/mL LAG3 antibody or IgG control. (**C**) Paired comparisons of AKT, mTOR, and S6 phosphorylation after treatment with the above antibody in NK cells from the HIV group (*n* = 7). (**D**) Paired comparisons of IFN-γ production by NK cells from the HC (*n* = 6) and HIV (*n* = 6) groups after 24 h stimulation with 2-DG, glucose, and the cytokine mixture. (**E**) Paired comparisons of IFN-γ–producing LAG3+ and LAG3− NK cells from the HC (*n* = 8) and HIV (*n* = 6) groups after 24 h stimulation with glucose and the above cytokine mixture.

Based on the results of the abovementioned transcriptomic analysis, we hypothesized that LAG3 activation downregulates glycolysis in NK cells by inhibiting the transcription of PI3K/AKT-regulated mechanistic target of rapamycin (mTOR) signaling, MYC, and several glycolysis-related enzymes (Fig. 5A). To test this hypothesis, we examined the NK cell surface expression of Glut-1 and the phosphorylation levels of intracellular AKT, mTOR, and S6 after LAG3 activation *in vitro*. Glut-1 expression, as depicted in Fig. 5B, and the levels of AKT, mTOR, and S6 phosphorylation, illustrated in Fig. 5C, underwent significant downregulation upon LAG3 activation within the HIV group. Inhibition of glycolysis through the administration of 2-deoxyglucose (2-DG) suppressed the production of IFN-γ in NK cells. Notably, this suppression was partially mitigated by the supplementary addition of exogenous glucose in both the HIV and HC groups, as depicted in Fig. 5D. In the absence of exogenous glucose, LAG3+ NK cells produced lower levels of IFN-γ than LAG3− NK cells (Fig. 5E) in HIV and HC groups. Administration of exogenous glucose reversed the dysfunction of LAG3+ NK cells (Fig. 5E), such that they did not differ significantly from LAG3− NK cells. These results clearly demonstrate for the first time that LAG3 activation leads to the inhibition of glycolysis via the PI3K/AKT/mTOR signaling pathway, which in turn suppresses the function of NK cells.

### Activated LAG3 suppressed the STAT1/IFNG pathway by upregulating PSAT1 expression

**Fig 6:**
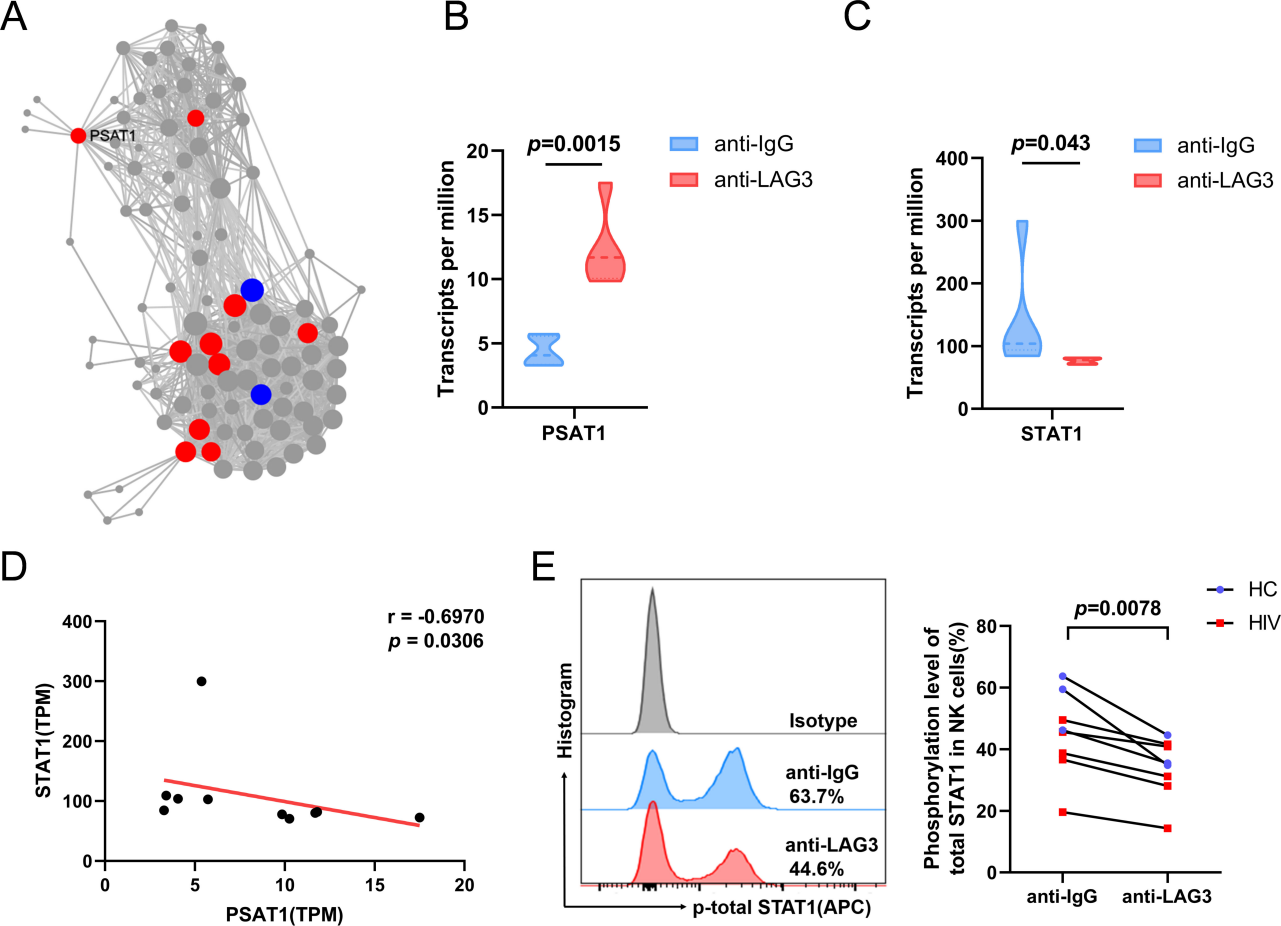
Transcriptome sequencing and *in vitro* analyses showed that activated LAG3 suppresses the STAT1/IFNG pathway by upregulating PSAT1 expression. (**A**) Gene subnetworks and top network key drivers of upregulated DEGs. (**B and C**) Paired comparisons of PSAT1 and STAT1 TPM expression in NK cells from the anti-LAG3 (*n* = 5) and anti-IgG (*n* = 5) groups. (**D**) Correlation analysis of PSAT1 and STAT1 TPM expression in NK cells (*n* = 10). (**E**) Paired comparisons of total STAT1 phosphorylation after treatment with 10 µg/mL LAG3 antibody or IgG control in NK cells from the HC (*n* = 3) and HIV (*n* = 5) groups.

Subsequent KDA of significantly upregulated genes (Fig. 6A) identified the phosphoserine aminotransferase 1 (PSAT1) gene as one of the top 10 key genes in the molecular connection network. The expression of PSAT1 was upregulated (Fig. 6B) and that of STAT1 significantly downregulated (Fig. 6C) in anti-LAG3-treated NK cells. Notably, the expression of STAT1 and PSAT1 was negatively correlated (Fig. 6D). Similarly, another study showed that overexpression of PSAT1 promotes the metastasis of lung adenocarcinoma via the inhibition of STAT1 as well as its downstream targets, IRF1 and IFNG (15). Our *in vitro* analyses confirmed reduced phosphorylation of STAT1 in NK cells treated with anti-LAG3 antibody in both HIV and HC groups (Fig. 6E). In summary, our data indicate that LAG3 activation in NK cells leads to PSAT1 overexpression and subsequent inhibition of IFN-γ production via the STAT1/IFNG pathway.

## DISCUSSION

We demonstrated that LAG3 expression on NK cells is upregulated in HIV groups, and we observed a negative correlation between LAG3 expression and the absolute CD4^+^ T cell count and CD4/CD8 ratio. In an earlier mouse study, Miyazaki et al. reported LAG3 expression on poly I:C-stimulated mouse NK cells (16). *In vitro* stimulation with IL-15 and NKG2C ligation was shown to induce LAG3 expression on NK cells isolated from human cytomegalovirus (HCMV)-seropositive donors (17). Similarly, LAG3 expression on healthy donor NK cells stimulated with IL-12 and IL-18 or IL-2 and IFN-α is upregulated (18, 19). The underlying cause of the elevated LAG3 expression on NK cells during HIV infection is still unclear; however, we speculate that it may be associated with the activation of the immune system. Taborda et al. reported that the immune system of HIV progressors is in a hyperactivated state, with higher expression of LAG3 on NK cells compared to HIV elite controllers, suggesting that individuals exhibiting failure in viral control have higher basal expression of markers associated with immune exhaustion, consistent with our results (20). Our comprehensive analysis of three NK cell subsets showed that CD56bright NK cells expressed higher levels of LAG3 in HIV groups. CD56^bright^ NK cells exhibit strong proliferation and differentiation capability and produce more IFN-γ (21), which plays an important role in immune regulation. Therefore, we hypothesize that the higher LAG3 expression in this subset negatively regulates the immune response by inhibiting NK cell proliferation, maturation, and antiviral cytokine production, thereby compromising the ability of NK cells to control HIV infection. However, we also acknowledge that LAG3, as a marker characterized by a continuum of expression, maybe more aptly represented by the MFI rather than frequency when it comes to depicting its expression level.

Our experimental observations confirm the abovementioned hypothesis. We found a significant reduction in IFN-γ production by LAG3+ NK cells from HIV groups, and NK cell proliferation and IFN-γ production were significantly upregulated after LAG3 ligand blockade using recombinant LAG3-Fc protein. In mouse model experiments, knockout of the LAG3 gene rendered NK cells unable to kill specific tumor targets (16). Experiments conducted with human NK cells yielded contrasting findings, as LAG3+ NK cells exhibited reduced IFN-γ production upon stimulation with K562 target cells (17). Narayanan et al. reported that LAG3 blockade results in enhanced human NK cell production of MIP-1α, IFN-γ, TNF-α, and MIP-1β *in vitro* upon stimulation with THP1 target cells (18). The findings from these studies support the results of our study indicating that LAG3 exerts a negative regulatory function on NK cell cytokine production during HIV infection.

Moreover, we observed marked downregulation of several glycolysis-related regulatory genes and the phosphorylation of AKT, mTOR, and S6 in NK cells after LAG3 activation. Moreover, we found downregulation of Glut-1 expression on NK cells, suggesting that LAG3 inhibits glycolysis in NK cells via the PI3K/AKT/mTOR signaling pathway, thus affecting NK cell function.

Changes in certain metabolic pathways are known to regulate NK cell activation and function; thus, regulating metabolic activity may be the key to releasing the full potential of NK cells (22). Previous studies have confirmed that 2-DG, a glucose metabolism inhibitor, suppresses the proliferation and cytotoxicity of IL-15-activated NK cells *in vitro* (23), which also supports our findings. The PI3K/AKT/mTOR signaling pathway regulates glycolysis by upregulating GLUT1 and GLUT4 expression, thereby enhancing glucose uptake and modulating the activity or expression of key glycolytic enzymes, including HK2, PFK1, and PFK2, thus playing an important role in glucose metabolism in cancer cells (22, 24, 25). S6, as a representative mTORC1 protein, is the core protein that regulates metabolic reprogramming to produce the key effector molecules granzyme B and IFN-γ in NK cells (26). Dana et al. found that knockout of the LAG3 gene or blockade of LAG3 *in vivo* leads to upregulation of glycolysis in immature CD4^+^ T cells, and they demonstrated that LAG3 regulates STAT5 and Akt activation (27). Their data support our findings in NK cells. PI3K is regulated by a variety of upstream regulators, such as Janus kinase, RAS proteins, receptor tyrosine kinases, and G protein-coupled receptors (28–31). The intracellular region of LAG3 comprises three components: a serine phosphorylation site, a “KIEELE” motif which is essential for LAG3-mediated inhibition of CD4^+^ T cells, and a glutamate-proline dipeptide repeat sequence. Nevertheless, LAG3 lacks a typical inhibitory motif, such as an immunoreceptor tyrosine-based inhibitory motif (32–35). Unfortunately, the mechanism of signal transduction involving LAG3 and PI3K remains unclear and should be explored further in future studies.

We found that the activation of LAG3 upregulates PSAT1 expression. PSAT1 is a key enzyme in the conversion of intermediate 3-PG to serine in the glycolysis shunt (36). We hypothesize that the upregulation of PSAT1 expression promotes the flow of more glycolysis intermediates into the serine synthesis pathway, which inhibits the main energy source pathway of NK cells and results in a decrease in IFN-γ production by NK cells. Notably, STAT1 expression was inhibited when PSAT1 expression increased, with a significant negative correlation observed. Yung-Chieh et al. reported that overexpression of PSAT1 promotes the metastasis of lung adenocarcinoma via the inhibition of STAT1 and its downstream signaling molecules IRF1 and IFNG (15), consistent with our findings. STAT1 is an important regulator that mediates the maturation, production of IFN-γ, and cytotoxicity of NK cells. STAT1-deficient mice exhibit heightened susceptibility to bacterial and viral infections (37, 38), and human STAT1 deficiency results in an autosomal recessive immunological disease characterized by repeated bacterial and viral infections, indicating impaired NK cell activity (39–42). For this reason, we hypothesized that this may be another signaling mechanism of NK cell function that is inhibited by LAG3, but detailed elucidation of the mechanism will require further studies.

In addition to LAG3, NK cells express several inhibitory receptors, including the revolutionized CTLA-4, PD-1, and recently identified TIGIT, TIM-3, Siglec-7/9, CD200, and CD47 (10). TIGIT is the most well understood, and blocking TIGIT gives rise to both loss of NK inhibition and enhanced activation. Our team previously reported that HIV-infected individuals expressed more TIGIT molecules on NK cells, and TIGIT blockade was shown to enhance human NK cell functions (43). Also, CD47 and its ligands inhibit NK cell activation, proliferation, and function in HIV-infected individuals, which is reported by our team (44). While numerous inhibitory receptors have been identified, whether their expression is increased on NK cells in HIV-infected individuals, whether they exert a repressive effect, and which receptors play the main inhibitory role remain to be studied.

**Fig 7:**
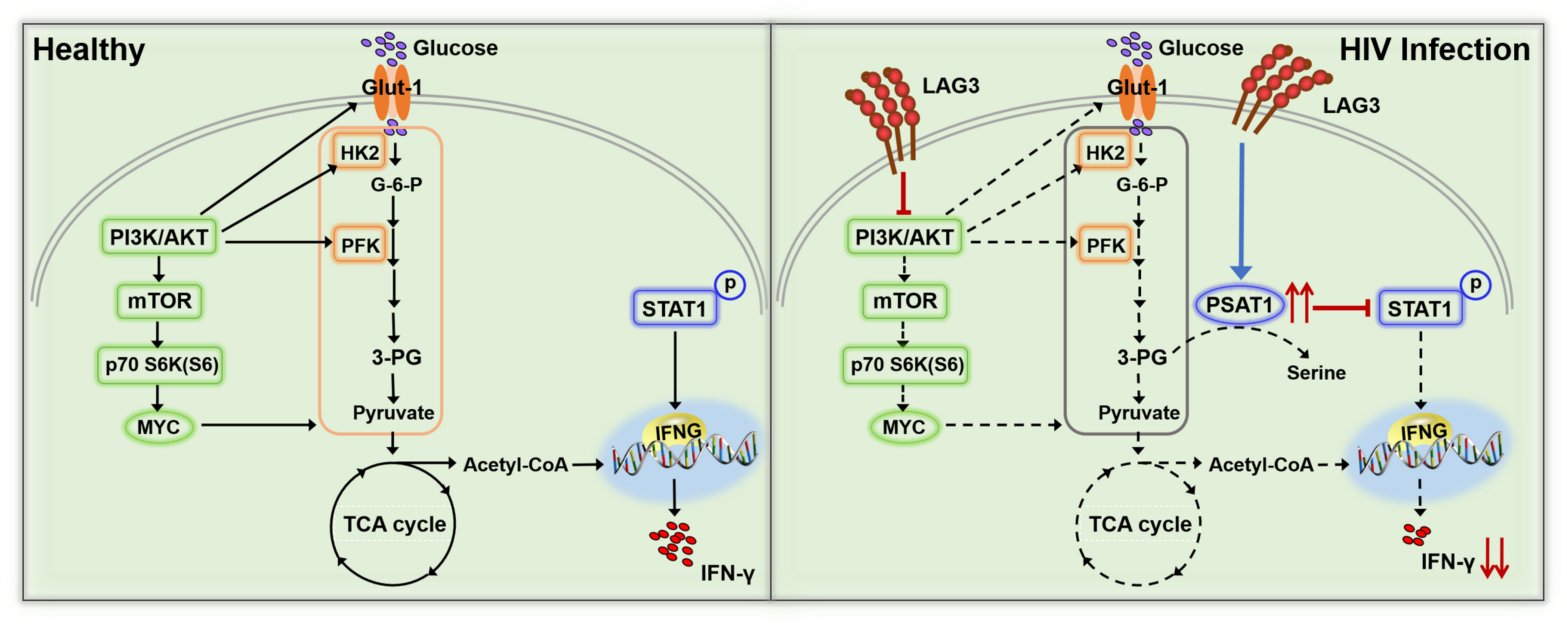
Schematic illustration of the mechanism of LAG3 inhibition of IFN-γ production by NK cells. During HIV infection, the expression of LAG3 on NK cells is increased, thereby activating the cells, which in turn inhibits the PI3K/AKT/mTOR signaling pathway regulating glycolysis, thus limiting NK cell IFN-γ production through a reduction in glycolysis. Simultaneously, LAG3 suppresses the STAT1/IFNG pathway by upregulating PSAT1 expression, which in turn suppresses IFN-γ production in NK cells.

Collectively, our results showed that LAG3 expression is upregulated on NK cells in HIV groups and negatively correlated with the CD4/CD8 ratio and CD4^+^ T cell count. LAG3+ NK cells exhibit reduced activation, proliferation, and IFN-γ production. Our transcriptome sequencing and *in vitro* analyses found that on the one hand, LAG3 inhibits glycolysis in NK cells glycolysis, and on the other hand, LAG3 suppresses activation of the STAT1/IFNG pathway by upregulating PSAT1 expression, thus restricting IFN-γ production by NK cells (Fig. 7). These findings indicate that LAG3 might be a useful novel target for immunotherapies to restore NK cell function during HIV infection.

## MATERIALS AND METHODS

### Study participants

This study included a total of 195 participants, in which there are 54 HIV-negative HCs who were also negative for hepatitis B and hepatitis C virus and had no diseases of the immune system, 141 PLWH, among whom 25 had not received antiretroviral therapy (HIV ART−) and 116 who were ART-treated (HIV ART+, also referred to as the HIV group) with an undetectable viral load on treatment for over 2 years. HIV-positive participants were recruited from the Clinic of the First Hospital, China Medical University.

### Detection of NK cell surface markers and nuclear marker Ki67

Peripheral blood mononuclear cells (PBMCs) were freshly isolated and surface stained with CD3-FITC, CD14-PerCP, CD19-PerCP, CD16-APC-Cy7, CD56-PE-Cy7, LAG3-PE or LAG3-BV421, CD38-BV421, HLA-DR-APC, CD69-PE (BioLegend), NKG2C-APC, and Glut-1-PE (R&D Systems) antibodies to analyze the expression of LAG3, NKG2C, CD38, HLA-DR, CD69, and Glut-1 on NK cells. After surface staining, the reagents from Thermo Fisher for intracellular fixation and permeabilization and Ki67-BV421 or Ki67-APC (BioLegend) were used to stain for the nuclear marker Ki67. Sample analysis was carried out with the BD FACS Canto II cytometer (BD Biosciences) and FlowJo 10.5.3 software.

### NK cell IFN-γ production assays

For stimulated groups, PBMCs were stimulated with the cytokine mixture, including IL-12 (10 ng/mL), IL-15 (50 ng/mL), and IL-18 (100 ng/mL), from R&D Systems at 37°C with 5% CO_2_ for 24 h. For unstimulated groups, PBMCs were cultured without the cytokine mixture. For the final 4 h, GolgiStop (BD Biosciences) was added to the culture. The cells were then collected and stained with Fixable Viability Stain 620 to exclude dead cells and then stained for surface markers (CD16-APC-Cy7, CD56-PE-Cy7, CD3-FITC, CD19-PerCP, CD14-PerCP, LAG3-PE, or LAG3-BV421). Fixation and permeabilization (reagents from BD Biosciences) were followed by intracellular staining with IFN-γ-APC (BioLegend).

### Assays of LAG3 ligand blockade

Recombinant human LAG3 Fc Chimera Protein or IgG Fc protein (R&D Systems) were added to PBMCs and incubated for 24 h, and the expression levels of CD69, HLA-DR, NKG2C, CD38, and Ki67 were evaluated as described above. For detection of IFN-γ production, LAG3 Fc and IgG Fc protein were added to PBMCs for 2 h before stimulation with the above cytokine mixture, and IFN-γ production was assessed using the method described earlier.

### Cytotoxicity assay

Cytotoxicity assays, employing flow cytometry, were conducted with K562 cells as the target and NK cells, treated with either recombinant human LAG3 Fc Chimera Protein or IgG Fc protein, serving as the effector cells. In a 96-well plate, PBMCs from healthy controls were incubated with either LAG3 Fc Chimera Protein or IgG Fc protein. After a 24 h co-incubation period, carboxyfluorescein succinimidyl ester (CFSE)-labeled K562 target cells were introduced to the wells, achieving an effector-to-target ratio of 30:1. These plated K562 targets, and effector NK cells were then co-incubated for an additional 6 h at 37°C, in an atmosphere containing 5% CO_2_. Subsequently, the cells were stained with 7-AAD (eBioscience, San Diego, CA, USA). To determine cytotoxicity, CFSE-positive K562 cells were gated, and the percentage of these cells that were also stained with 7-AAD was calculated.

### Analysis of the effect of 2-DG and exogenous glucose on IFN-γ production of NK cells

PBMCs were seeded and pre-treated with 2-DG (5 mmol/L; Sigma) and exogenous glucose (10 mmol/L; Sigma) for 2 h before stimulation with cytokine mixture. IFN-γ production was evaluated as described above.

### Analysis of signaling pathway phosphorylation levels

Negative isolation of NK cells from PBMCs was performed with the EasySep Human NK Cell Isolation kit (Stemcell). The sorted NK cells were stimulated with the cytokine mixture for 15 minutes and then immediately treated with BD Fix Buffer I in a 37°C water bath for 10 minutes and Perm Buffer III (BD Biosciences) on ice for 30 minutes, followed by staining with the phosphorylation markers AKT-FITC, mTOR-PE, total STAT1-APC (BD Biosciences), and S6-APC (eBioscience). BD FACS Canto II flow cytometry was used to assess the phosphorylation of AKT, mTOR, S6, and STAT1 in NK cells.

### Analysis of LAG3 activation

Purified anti-human LAG3 antibody (5, 10, and 20 µg/mL) or purified mouse IgG κ isotype control (10 µg/mL) from BioLegend was added to PBMCs or sorted NK cells and incubated for 2 h before stimulation with the cytokine mixture. After incubation, the expression of Glut-1, Ki67, and IFN-γ was evaluated as described above; the sorted NK cells were incubated with 10 µg/mL LAG3 antibody and 10 µg/mL IgG control for 2 h. The phosphorylation of AKT, mTOR, S6, and STAT1 in NK cells was evaluated as described above. The treated NK cells with LAG3 antibody and IgG control for 24 h were used for transcriptome analysis.

### Transcriptome sequencing analysis

The DNBSEQ platform of BGI was used to analyze the transcriptome. Differential expression analysis was carried out using DESeq2 with a *Q* value <0.05. Heatmaps were drawn using Phatmap and the Complex Heatmap R package. Enrichment analyses for KEGG pathways and GO biological processes were conducted on annotated differentially expressed genes using Phyper. Transcriptome GSEA analysis was performed using GSEA v3.0 software. The H (hallmark) and C5 (GO) biological process data sets in the MSigDB data set were used for enrichment analyses; the significance of enrichment was determined based on a false discovery rate < 0.25 and a *P*-value < 0.05.

### Quantification and statistical analysis

Statistical analyses and graph production were carried out with SPSS 26.0 and GraphPad Prism 8.0 software. For paired data, in the case of normally distributed data, the paired *t*-test was applied to analyze differences; otherwise, the Wilcoxon matched-pairs signed-rank test or Friedman tests with Dunn’s multiple comparisons tests were applied. For unpaired and non-normally distributed data, the nonparametric Mann-Whitney *U* test was applied to evaluate differences between two groups, and the Spearman’s rank test was used to analyze the correlation between two indexes. Statistical significance was defined as *P* < 0.05 (two sided).

## SUPPLEMENTAL MATERIAL

Supplemental figures mbio.00230-25-s0001.pdf Figures S1 and S2.

ASM does not own the copyrights to Supplemental Material that may be linked to, or accessed through, an article. The authors have granted ASM a non-exclusive, world-wide license to publish the Supplemental Material files. Please contact the corresponding author directly for reuse.
