# Supplementary material for: The inhibitory receptor LAG3 affects NK cell IFN-γ production through glycolysis and the PSAT1/STAT1/IFNG pathway
Source: mBio. 2025 Apr 29;16(6):e00230-25. doi: 10.1128/mbio.00230-25 (PMC12153268; doi:10.1128/mbio.00230-25)
Supplement: Supplemental figures — Figures S1 and S2. [file mbio.00230-25-s0001.pdf]

Supplemental information

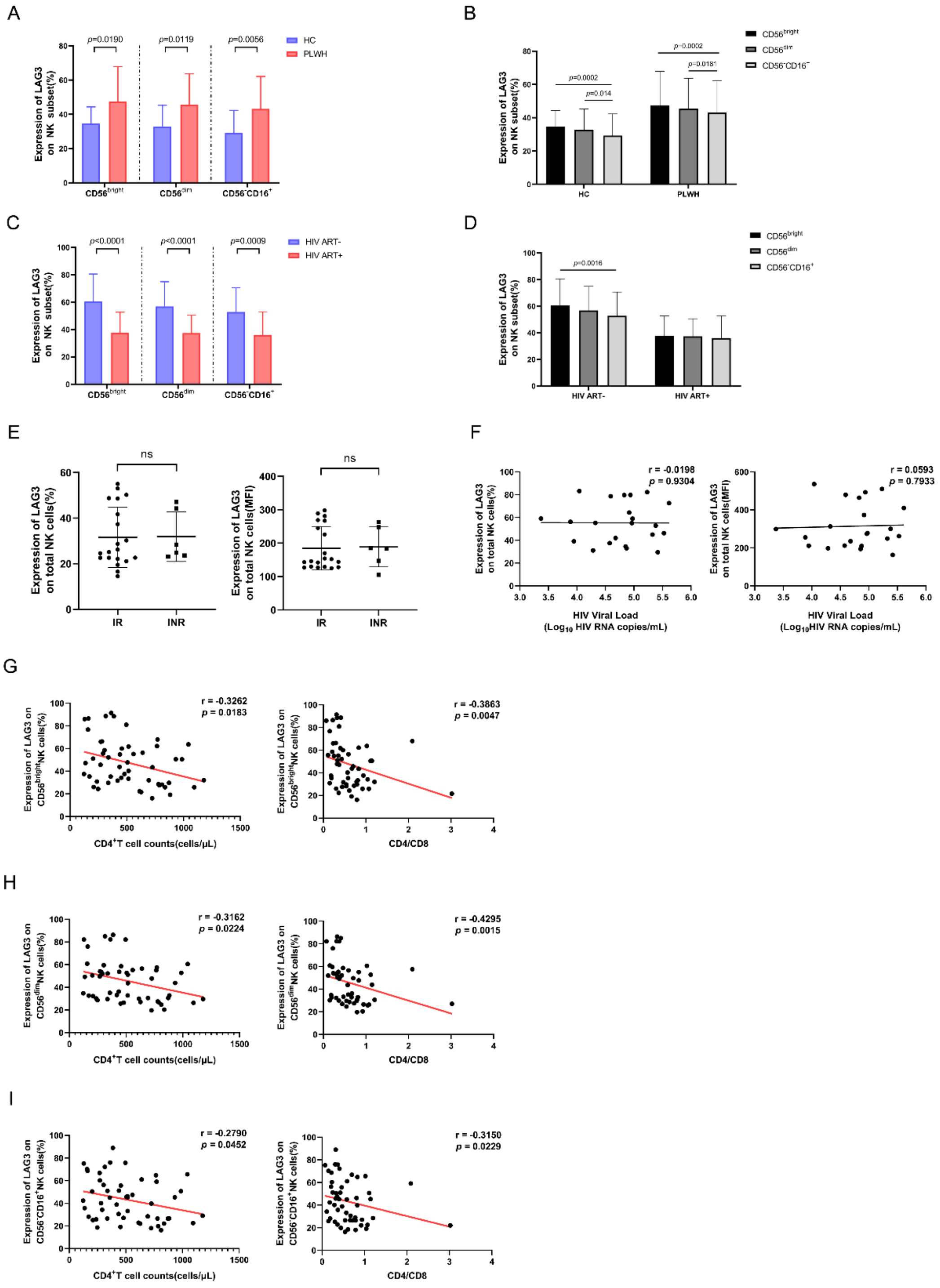

Figure S1. Expression of LAG3 on NK cell subsets in PLWH and HC groups and correlation with HIV disease progression.

(A, C) Comparison of the percentage of LAG3 expressed on various NK cell subsets between the HC (n = 25) and PLWH (n = 52) groups, HIV ART−(n = 22) and HIV ART+ (n = 30) groups. **(B, D)** Within-group comparison of the percentage of LAG3 expressed on different NK cell subsets in the HC (n = 25) and PLWH (n = 52) groups, HIV ART− (n = 22) and HIV

ART+ (n = 30) groups. All HIV ART+ groups had an undetectable viral load. **(E)** Comparison of the percentages (left) and MFI (right) of LAG3 on total NK cells between the IR (n = 20) and INR (n = 6) from HIV ART+ groups. **(F)** Analysis of the Spearman correlation between LAG3 expression on NK cells (left: percentage; right: MFI) and plasma level of HIV Viral Load in the HIV ART− (n = 22) group. **(G-I)** Spearman correlation analysis between LAG3 expression on CD56bright, CD56dim, CD56-CD16+ NK cells and absolute CD4^{+} T cell count (cells/μL) (left) and CD4/CD8 ratio (right) (n = 52).

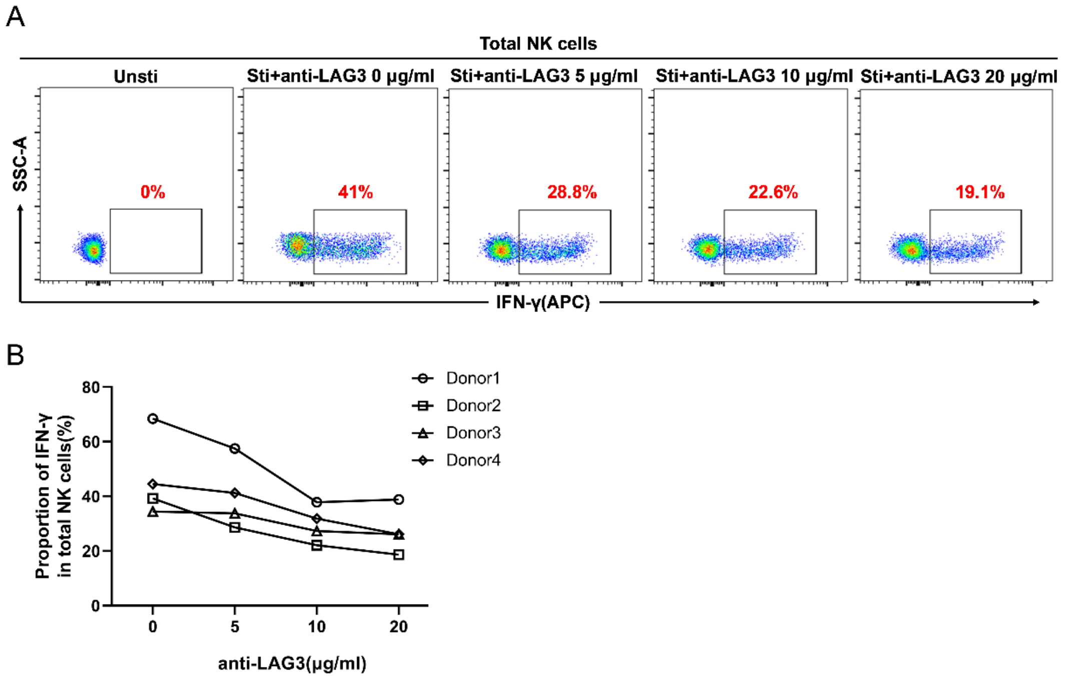

Figure S2. Effect of different doses of anti-LAG3 antibody on IFN-γ production in NK cells.

(A) Representative flow cytometry plots demonstrating the effect of different doses of anti-LAG3 antibody (0 [medium only], 5, 10 and 20 μg/mL) on IFN-γ production in NK cells. **(B)** Proportion of IFN-γ+ NK cells in HIV groups following treatment with varying concentrations of anti-LAG3 antibodies (0, 5, 10, and 20 μg/mL; n = 4).
